# Increased Arf/p53 activity in stem cells, aging and cancer

**DOI:** 10.1111/acel.12574

**Published:** 2017-01-19

**Authors:** Estefania Carrasco‐Garcia, Manuel Moreno, Leire Moreno‐Cugnon, Ander Matheu

**Affiliations:** ^1^ Cellular Oncology GroupBiodonostia InstituteSan SebastianSpain; ^2^ IkerbasqueBasque FoundationBilbaoSpain

**Keywords:** aging, ARF, p16, p53, stem cells

## Abstract

Arf/p53 pathway protects the cells against DNA damage induced by acute stress. This characteristic is the responsible for its tumor suppressor activity. Moreover, it regulates the chronic type of stress associated with aging. This is the basis of its anti‐aging activity. Indeed, increased gene dosage of Arf/p53 displays elongated longevity and delayed aging. At a cellular level, it has been recently shown that increased dosage of Arf/p53 delays age‐associated stem cell exhaustion and the subsequent decline in tissue homeostasis and regeneration. However, p53 can also promote aging if constitutively activated. In this context, p53 reduces tissue regeneration, which correlates with premature exhaustion of stem cells. We discuss here the current evidence linking the Arf/p53 pathway to the processes of aging and cancer through stem cell regulation.

## Ink4/Arf/p53 activity in cancer

Cancer is the consequence of an aberrant gain of cellular fitness linked to the accumulation of stress and cellular damage of acute intensity. This damage occasionally provides aberrant advantages to certain cells, which can eventually lead to cancer development. The *Ink4/Arf* locus and *p53* are regarded as the most relevant tumor suppressors based on their ubiquitous and frequent inactivation in human cancer. The *Ink4/Arf* locus encodes three tumor suppressor genes *p15* ^*Ink4b*^ *, p16* ^*Ink4a*^ *,* and *p14* ^*Arf*^ (p19^Arf^ in mice). On one hand, p15^Ink4b^ and p16^Ink4a^ (called Ink4 hereafter) inhibit the formation of the cyclin‐dependent kinases (CDK4 and CDK6) and cyclinD complexes during the G1 phase of the cell cycle. Hence, they prevent the transcription of genes involved in the transition to S phase, importantly the Rb/E2F1 pathway, so regulating cell cycle progression (Yaswen *et al*., 2015). On the other hand, Arf exerts its tumor suppressive action by inhibiting Mdm2, a ubiquitin ligase considered the major p53 regulator, thereby contributing to the activation and stabilization of p53 (Matheu *et al*., 2008).

**Figure 1:**
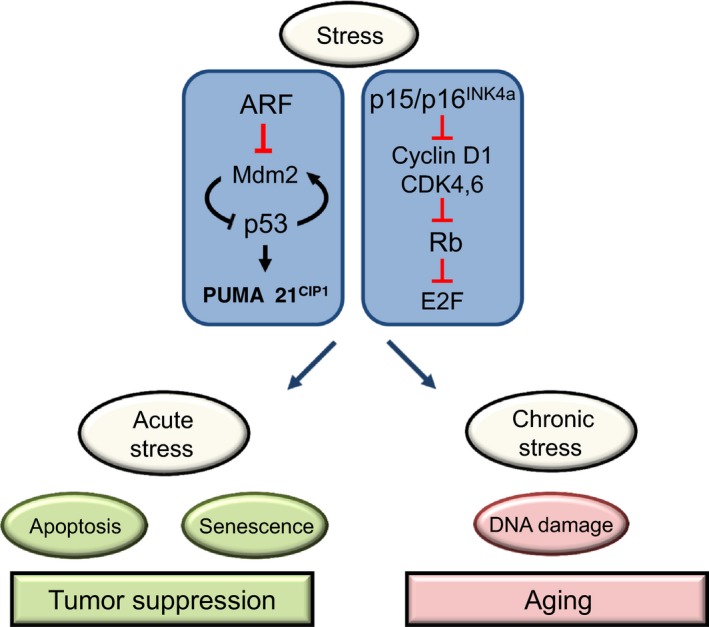
*Ink4/Rb‐* and *Arf/p53*‐mediated responses to cellular stress. In response to stress, cells activate *Ink4/Rb* and *Arf/p53* signaling pathways in order to restore the homeostasis or eliminate themselves, preventing tumor formation in the case of acute oncogenic stress or alleviating accumulated cellular damage produced by chronic stress related to aging. p16^Ink4a^ blocks the cell cycle though its inhibitory action on the CDK/Cyclin D complexes, finally avoiding the E2F transcriptional action and the progression of cells from G1 to S phase. Arf promotes the degradation of the negative p53 regulator Mdm2, inducing p53 activation and, in consequence, p21‐mediated cell cycle arrest or senescence and/or PUMA‐mediated apoptosis.

The Ink4/Rb and Arf/p53 pathways are major sensors of stress that play a crucial role in early detection and elimination of cells that have suffered different types of stress including oncogene activation, DNA damage, oxidative stress, etc. While the activation of Ink4/Rb pathway induces reversible cell cycle arrest or irreversible cellular senescence‐associated changes, the activation of p53 elicits a cellular response that might vary from restoration of cellular homeostasis by a transient blockade of the cell cycle to allow for DNA repair, senescence, or apoptosis (Fig. 1). The activation of these responses depends in a complex manner, on the intensity of the triggering stress and on the cellular context. In agreement with this damage protective role, the individual or combinatory deletion of these genes promotes cancer susceptibility in multiple tissues and contexts. On the contrary, enhanced Ink4/Arf and p53 activity preserves mice from spontaneous or chemically induced cancers (Garcia‐Cao *et al*., 2002; Tyner *et al*., 2002; Maier *et al*., 2004; Matheu *et al*., 2004, 2007, 2009; Mendrysa *et al*., 2006).

## Ink4/Arf/p53 activity in aging

Aging is characterized by a loss of fitness, which results from the accumulation of cellular damage induced by chronic stress of small intensity. Moreover, there is clinical evidence that tumors have a higher incidence in aged organisms, which establishes a relationship between accumulated (likely pathogenic) cell damage, aging, and cancer development. Thus, although cancer and aging may seem opposite processes, they can be regarded as two different manifestations of the same underlying process, namely the accumulation of cellular damage. Moreover, cancer and aging may share common origins (Lopez‐Otin *et al*., 2013). There are several genetic or pharmacological manipulations that simultaneously modulate cancer and aging. For example, systemic downregulation of IGF1 signaling pathway by the overexpression of PTEN tumor suppressor increases longevity, delays aging, and confers cancer protection in mice (Garcia‐Cao *et al*., 2012; Ortega‐Molina *et al*., 2012). Similarly, reduced expression of c‐*Myc* oncogene increases lifespan and shows resistance to several age‐associated pathologies, such as osteoporosis, cardiac fibrosis and immunosenescence (Hofmann *et al*., 2015). Caloric restriction also protects from cancer and aging (Lopez‐Otin *et al*., 2013), whereas metformin and rapamycin, two pharmacological compounds, which concomitantly extend longevity and impair cancer formation and growth (Blagosklonny, 2014). These proofs demonstrate that cancer protection and longevity can be simultaneously modulated using different strategies and molecular mechanisms.

In recent years, it is deepening the knowledge of the implications that the Ink4/Rb and Arf/p53 pathways have on the management of cellular damage associated with the aging process. The observation that several manipulations simultaneously modulate longevity and cancer protection establishes an interesting parallel with the expression of members of the Ink4/Rb and Arf/p53 pathways, which are silent or very low during development and postnatal life, while progressively increase from adulthood to old age in a broad range of tissues and species (Zindy *et al*., 1997; Krishnamurthy *et al*., 2004). Moreover, human genomewide association studies have identified genetic variants in the *INK4/ARF* locus on 9p21.3 that confer increased risk of atherosclerotic vascular diseases such as stroke, aortic aneurysm or myocardial infarction, as well as to additional age‐related diseases such as type 2 diabetes or glaucoma (Liu *et al*., 2009; Jeck *et al*., 2012). Similarly, there is evidence of the association of single nucleotide polymorphisms located on this genomic region to exceptional longevity in centenarians although with contradictory results (Pinos *et al*., 2014; Congrains *et al*., 2015). These data confirm that the response to stress mediated by the *Ink4/Arf* locus is involved in age‐related pathologies. With regard to p53 in human aging, there is evidence that individuals carrying a common polymorphic variant of p53 (proline instead arginine in codon 72: p53‐Pro72), that is more active inducing cell cycle arrest and senescence rather than in apoptosis, present increased lifespan (van Heemst *et al*., 2005). Moreover, this polymorphism has implications in type 2 diabetes, wherein it is associated with a better metabolic status (Bonfigli *et al*., 2013). There is little additional information regarding p53 and aging in human, yet there is no evidence of a pro‐aging function. Indeed, it has been documented that p53 is not involved in human premature aging disorders such as Hutchinson–Gilford Progeria (O'Neill *et al*., 2003), and it has been postulated that well‐preserved p53‐mediated responses are likely a key factor contributing to protection from diseases and cancer in centenarians (Salvioli *et al*., 2009). In line with this, p53 anti‐aging activity, rare alleles of two exon‐derived SNPs of *p21* ^*CIP*^, well‐established p53 downstream target, are significantly underrepresented among the centenarians (Gravina *et al*., 2009). Previous studies have shown that the presence of these rare SNPs increases the susceptibility for the development of some types of cancer (Facher *et al*., 1997; Li *et al*., 2005). Thus, these p21^CIP^ alleles may be potentially detrimental to longevity and cancer protection.

The above raises the possibility that Ink4/Rb and Arf/p53 pathways might have a role in aging. Thus, stress conditions cause an accumulation of DNA damage at the cellular level. Ultimately, it leads to the final activation of the Ink4/Rb and Arf/p53 pathways in order to achieve various adaptive responses to this situation. Amidst such responses is the transient block of the cellular cycle to try to repair the damage, inducing a state of senescence, or even apoptosis. Therefore, the empowerment of Ink4/Rb and Arf/p53 pathways might play an important role not only on surveillance and suppression of tumors, but also on the accumulation of cellular damage and aging. Therefore, it is reasonable to surmise that Ink4/Rb and Arf/p53 play a role also in the response to age‐associated chronic stress and consequently affects aging. As activation of the Ink4/Rb and Arf/p53 pathways triggers a protective mechanism against tumor‐induced stresses, they could also have anti‐aging activity by alleviating the load of age‐associated damage (Fig. 1). However, the pleiotropic antagonism theory, which suggests that certain cellular processes that provide benefit in youth, may compromise organismal fitness in later life, postulates that tumor suppressors might have dual effects depending on the etiology of the cellular stress and the cellular and molecular context (Campisi, 2003).

The evaluation of the impact of the absence of *Ink4/Arf* locus and p53 on aging has not been studied nowadays, as null or heterozygous mice for these tumor suppressors develop several type of tumors at an early age (Donehower *et al*., 1992; Serrano *et al*., 1996), but it would be feasible in an inducible knockout or knockdown system. However, different mouse models with increased Ink4/Arf and p53 activity have been described in the last years, which allowed the understanding of the role of these tumor suppressors in aging and provided evidence supporting these two nondiscriminatory actions.

## Deregulated increase in Ink/Arf/p53 activity promotes premature aging

**Figure 2:**
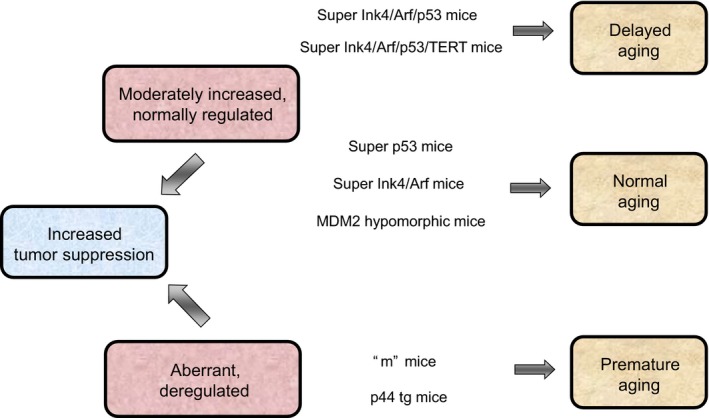
Mouse models with regulated or deregulated *Ink4/Rb* and/or *Arf/p53* pathways and their phenotypes with regard to tumor prevention and aging. Mutant mice with deregulated p53 hyperactivity display reduced tumor development but present premature aging and aging‐related diseases. Mice presenting a modest increase in normally regulated Ink4/Arf and p53 exhibit an enhanced tumor suppression capacity and normal aging. Mice presenting modest and regulated increases in both *p53* and *Ink4/Arf* in the absence or presence of a constitutive telomerase reverse transcriptase (TERT) display resistance to tumor formation and present delayed aging. ‘m’ mice: Mice expressing a constitutively active truncated form of p53 lacking exons from 1 to 6 (Tyner *et al*., 2002); p44 tg mice: Mice presenting a constitutively active truncated form of p53 lacking exons from 1 to 3 (Maier *et al*., 2004), super p53 mice: Mice harboring an extra copy of the p53 intact gene (Garcia‐Cao *et al*., 2002); super Ink4a/Arf mice: mice with an extra intact *Ink4/Arf* locus (Matheu *et al*., 2004); MDM2 hypomorphic mice: mice with reduced Mdm2 levels (Mendrysa *et al*., 2006); ‘super Arf/p53’ mice: mice harboring an extra copy of p53 and *Ink4/Arf* (Matheu *et al*., 2007); ‘super Arf/p53/TERT’ mice: super Arf/p53 mice additionally presenting constitutive telomerase reverse transcriptase (TERT) (Tomas‐Loba *et al*., 2008).

The pro‐aging function of deregulated p53 activity has been documented in two independent mutant mouse models (Fig. 2). Tyner *et al*. (2002) developed a mutant mouse model called ‘*m’* mice by deletion of the first six exons of the *p53* gene. The mutant form consisting in a carboxy terminal p53 fragment of 24 KDa lacks the Mdm2‐binding domain and, as a consequence, evades degradation by the proteasome and constitutively activates the endogenous wild‐type p53 and downstream effectors. These mutant mice display higher resistance to tumor development compared to control animals but present a shorter lifespan and premature aging, including various diseases related to aging, such as osteoporosis and early and intense loss of cellularity and tissue mass (Tyner *et al*., 2002), reflecting defects in organ homeostasis. In an additional study, Maier *et al*. (2004) developed another mouse model expressing a shorter *p53* isoform lacking exons from 1 to 3 that produced a truncated and Mdm2‐insensitive p53 protein of 44 KDa called *p44tg*. These mutant animals show increased resistance to spontaneous carcinogenesis but present a reduction in life expectancy and premature development of diseases such as osteoporosis and tissue atrophy. These effects are consequence of alterations in the signaling cascade of IGF affecting cell growth and proliferation. Moreover, they show a low rate of cellular replication resulting in small‐sized individuals with a shortened breeding period, which is more pronounced in males that are sterile before reaching one year (Maier *et al*., 2004).

Additional studies reinforce the pro‐aging function of deregulated p53 activity. Cao *et al*. show that the deficient activity of *BRCA1* and the consequent DNA damage stress cause increased senescence and premature aging in mice. Interestingly, these consequences are directly dependent on a strong activation of p53, as inferred from the fact that blocking its main effector, p21^CIP^, is enough to reverse the process (Cao *et al*., 2003). In the same line, Varela *et al*. noted that another model, in which permanent cell stress is induced by abnormalities in the nuclear structure due to the deficiency of the metalloproteinase Zmpste24, exhibits cellular senescence and premature aging. Notably, these processes are driven by p53 hyperactivation and the phenotype was partially reversed in a *p53*‐null genetic background (Varela *et al*., 2005).

## Normally regulated increase in Ink4/Arf/p53 activity in aging

Different researchers have generated genetically manipulated mice with a modest increase in normally regulated Ink4/Arf and p53 (Fig. 2). These include mice carrying transgenic alleles encoding the intact *p53* gene (Garcia‐Cao *et al*., 2002), the intact *Ink4/Arf* locus (Matheu *et al*., 2004), or mutant mice with reduced *Mdm2* activity (Mendrysa *et al*., 2006). In all these three models, the tumor suppressors retain their natural regulation, yet their levels are moderately increased. As example, those mice with additional regulated copies of *p53* or *Mdm2* inactivation present enhanced apoptosis induction in the thymus, revealing the expected p53 augmented activity (Garcia‐Cao *et al*., 2002; Mendrysa *et al*., 2006). Interestingly, all these models exhibit elevated tumor suppression potency, as are less prone to develop chemically or genetically induced sarcomas, adenomas or carcinomas, and present increased spontaneous cancer protection (Garcia‐Cao *et al*., 2002; Matheu *et al*., 2004; Mendrysa *et al*., 2006). Noteworthy, the proper regulation and moderate overexpression of the suppressors do not accelerate organismal aging and/or decrease longevity in any of the three models, providing a more potent and optimal response to chronic stress, along with cancer prevention, than p53 deregulated models. In agreement with the idea of enhanced and regulated p53 not affecting longevity and aging, the presence of the transgenic *p53* or *Ink4/Arf* allele in mice deficient in telomerase and apolipoprotein E‐null mice does not aggravate the accelerated aging or the atherosclerosis‐prone characteristic phenotypes of these mice (Garcia‐Cao *et al*., 2006; Sanz‐Gonzalez *et al*., 2007; Fuster *et al*., 2012). Similarly, iPS cells derived from *Ink4/Arf* or *p53* transgenic mice do not present any alteration in their pluripotency, but they had limited their tumorigenicity (Menendez *et al*., 2012). Notably, *Ink4/Arf* transgenic mice show a modest increase in their lifespan albeit not statistically significant (Matheu *et al*., 2009). In line with this result, the presence of an extra copy of the *Ink4/Arf* protects against insulin resistance and glucose intolerance, two syndromes associated with aging (Sanz‐Gonzalez *et al*., 2007). These results indicate that normally regulated activation of the Ink4/Rb and Arf/p53 pathways trigger a protective mechanism against tumor‐induced stresses and show that these genes might also have anti‐aging activity in some contexts by alleviating the load of age‐associated damage.

Interestingly, the combined effects of modest and regulated increases in *p53* and *Ink4/Arf* (*s‐Ink4/Arf/p53*) or two extra copies of *Ink4/Arf* (*s‐Ink4/Arf tg/tg*) result in a significantly elongated lifespan and delayed organismal aging (Matheu *et al*., 2007, 2009). The longevity of the transgenic mice is still longer than wild‐type mice when the survival analysis excludes the cases of cancer from both groups. Thereby, the Ink4/Rb and Arf/p53 pathways not only prevent cancer, but also contribute to attenuate the deleterious effects of aging (Matheu *et al*., 2007). Furthermore, these long‐lived phenotypes of *s‐Ink4/Arf/p53* mice are further potentiated in the presence of a constitutive telomerase reverse transcriptase (*s‐TERT*) (Tomas‐Loba *et al*., 2008). In support of the anti‐aging activity of p53, the elimination of p53 exacerbates the degenerative phenotype of different progeroid mouse models presenting alterations in telomere maintenance or DNA repair (Begus‐Nahrmann *et al*., 2009; Murga *et al*., 2009; Ruzankina *et al*., 2009). Moreover, the inhibition of the p53‐mediated apoptosis entails the formation of neoplasia and accelerates aging‐associated signs and reduces lifespan in *ATM* mutant mice (Armata *et al*., 2007).

We are beginning to understand the impact that the individual members of the Ink4/Rb and Arf/p53 pathways have on aging. Thus, the ablation of *p16* ^*Ink4a*^, but not *p19* ^*Arf*^, *p53,* or *p21* ^*CIP*^, alleviates the premature aging of mice with constitutively high levels of endogenous chromosome damage due to hypomorphic mutant alleles of *BubR1* (Baker *et al*., 2008, 2013). These results reveal an anti‐aging activity for the p53 pathway, likely attributable to the protection against aging‐accumulated oxidative and DNA damage (Matheu *et al*., 2007, 2009). Moreover, these works suggest that *p16* ^*Ink4a*^ might have pro‐aging activity. Additional evidences indicate that *p16* ^*Ink4a*^ might contribute to promote aging through its function as senescence inductor. Indeed, it has been shown that the clearance of *p16* ^*Ink4a*^ positive cells from the organism ameliorates aging and shortens healthy lifespan in mice (Baker *et al*., 2011, 2016). Furthermore, transgenic mice in which p16^Ink4a^ is conditionally overexpressed display strong inhibition of proliferation of normal intestinal cells in young mice and premature induction of several features of aging such as reduced hair density, variable lightening of hair color, lower weight, and kyphosis (Boquoi *et al*., 2015). However, this activity does not seem related to senescence as the induction of p16^Ink4a^ did not detectably increase senescence‐associated ß‐galactosidase staining in intestine and the de‐induction of p16^Ink4a^ revealed that the premature aging features were reversible (Boquoi *et al*., 2015). Additional evidence supports a more complicated picture of the role of p16^Ink4a^ on aging. Thus, the effects of a transgenic allele with constitutive and systemic overexpression of p16^Ink4a^ are minimal in the pancreatic islets and in the brain (Krishnamurthy *et al*., 2006; Molofsky *et al*., 2006), while a luciferase knockin mouse model (*p16* ^*LUC*^), which faithfully reports the expression of p16^INK4a^, revealed an exponential increase in luminescence with aging, but could not predict overall mortality and development of spontaneous malignancy (Burd *et al*., 2013). Alternatively, it has been also postulated the possibility that *p16* ^*Ink4a*^ is indeed an anti‐aging gene, through its capacity to slow down proliferation (Matheu *et al*., 2009). Moreover, there are some integration and cross talk between Ink4/Rb and Arf/p53 pathways that might be important when studying their function in aging. Thus, Arf/p53 pathway collaborates with p16^Ink4a^ to activate Rb through the induction of p21^cip^ expression (McConnell *et al*., 1999; Mitra *et al*., 1999). Moreover, inactivation of p16^Ink4a^ /Rb pathway induces upregulation of p14^Arf^ expression through the activation of E2F1 (Bates *et al*., 1998), whereas inactivation of p53 causes compensatory upregulation of p16^Ink4a^ expression (Leong *et al*., 2009; Yamakoshi *et al*., 2009).

## Increased Arf / p53 activity in stem cells

The fundamental manifestation of aging is an overall decline in the functional capacity of organs to maintain tissue homeostasis and respond adequately to physiological needs under conditions of chronic stress. The regenerative potential of tissues relies on the activity of stem cells, becoming the maintenance of stem cell quiescence and self‐renewal critical processes involved on tissue homeostasis. Thereby, stem cell exhaustion has been postulated as one of the ‘Hallmarks of aging’ (Lopez‐Otin *et al*., 2013). In line with this idea, stem cell rejuvenation is presented as a promising strategy to mitigate organismal aging. In the particular case of the central nervous system, the impairment of the brain is a common feature of normal aging, which coincides with a reduction in the number of neural stem/progenitor cells (NSCs), resulting in functional decline of the brain activity, cognitive impairment, and neurodegenerative diseases (Fuentealba *et al*., 2012).

**Figure 3:**
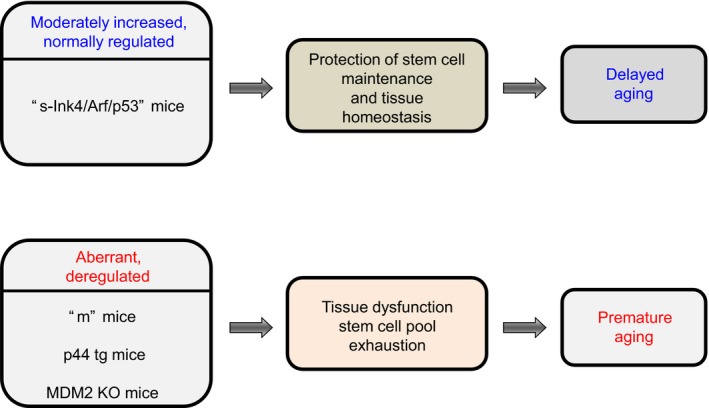
Ink4/Rb and Arf/p53 pathways action on stem cell niches and aging modulation. Mice models with deregulated hyperactivation of p53 exhibit stem cell exhaustion along with tissue homeostasis disruption that promotes premature aging. However, mice with an extra copy of Ink4a/Arf/p53 display an improved maintenance of stem cells that provides tissue homeostasis delaying aging.

The above raises the possibility that Ink4/Rb and Arf/p53 might play their role in aging through the modulation of the stem cell activity. Indeed, p53 deregulated premature aging mouse models present decreased cell replacement in tissues, which has been linked to a premature depletion of stem cell niches. Thereby, the ‘*m’* mouse model exhibits exhaustion of hematopoietic stem cells (HSCs) along with reduced hematopoiesis (Dumble *et al*., 2007), and also disruption in mammary gland morphogenesis attributable to the depletion of stem cells (Gatza *et al*., 2008). Similarly, adult *p44tg* mice present a reduced number of NSCs and impoverished olfactory capacities (Medrano *et al*., 2009). These mice also present an impaired replacement of beta cells in the pancreas and the consequent alteration of glucose homeostasis (Hinault *et al*., 2011). Accordingly to these findings, mice with p53 overactivation in the epidermis by Mdm2 ablation show diminished epidermal stem cell activity and skin prematurely aged (Gannon *et al*., 2011). These results indicate that deregulated p53 impacts on lifespan by a decline in tissue stem cell regenerative function likely as a consequence of premature stem cell exhaustion (Fig. 3).

On the contrary, the extended lifespan and delayed aging of mice with an extra copy of *Ink4a/Arf/p53* have been linked to an increased maintenance of stem cells pools. For example, s‐*Ink4/Arf/p53* aged transgenic animals present greater preservation of the capacity of proliferation and self‐renewal of NSCs compared to control littermates demonstrated by the relative increase in the number of neurospheres in culture and the higher expression of several markers of NSCs *in vivo* (Carrasco‐Garcia *et al*., 2015). This positive effect of extra *Ink4/Arf/p53* on the maintenance of NSCs directly impacts on mice behavior and neuronal activity, as revealed by enhanced functional activity in various behavioral tests such as neuromuscular coordination or cognitive performance (Carrasco‐Garcia *et al*., 2015). According with this evidence, *Ink4/Arf/p53* aged transgenic mice also display delayed epithelial stem cell exhaustion in the skin and improved hair regeneration compared to control mice (Matheu *et al*., 2007; Tomas‐Loba *et al*., 2008). These results confirm that modest and regulated increases in Ink4/Arf/p53 tumor suppressors result in systemic organismal benefits ameliorating stem cell aging and maintaining tissue homeostasis (Fig. 3). In this case, the anti‐aging activity of Arf/p53 is dependent on the conventional p53 pathway affecting its major role as regulator of cellular proliferation. Thus, moderate upregulation of the p53 pathway during aging likely results in slower proliferation rates, probably contributing to their quiescence and long‐term maintenance and delaying stem cell exhaustion. In support of this idea, *p53* or *p21* ^*CIP*^ inactivation results in loss of quiescence and exhaustion of long‐term stem cells at advanced ages (Cheng *et al*., 2000; Kippin *et al*., 2005; Meletis *et al*., 2006). Moreover, p53 also maintains the self‐renewal of the nephron progenitor pool in the mouse kidney (Li *et al*., 2015). With regard to the role of the p16^Ink4a^, there is no clear conclusion. In one hand, it negatively affects the maintenance of stem cell pools in the brain, hematopoietic system, or pancreas (Janzen *et al*., 2006; Krishnamurthy *et al*., 2006; Molofsky *et al*., 2006), and its inducible overexpression inhibits intestinal *Lgr5* ^*+*^ stem cell proliferation (Boquoi *et al*., 2015), supporting an *Ink4/Arf* pro‐aging activity. On the other hand, aged transgenic mice overexpressing specifically p16^Ink4a^ do not present any significant deleterious impact in NSC or pancreatic progenitors (Krishnamurthy *et al*., 2006; Molofsky *et al*., 2006).

*Ink4/Arf/p53* aged transgenic mice also present an increase in markers of neuroblasts (DCX and PSA‐NCAM) and in markers of new neurons (NeuN) in the olfactory bulb and dentate gyrus (Carrasco‐Garcia *et al*., 2015). Similarly, deletion of *p53* does not influence the HSC pool size in *Wip1* mutant mice but rescues their multilineage repopulation defect (Chen *et al*., 2015), together indicating that p53 might also regulate stem cell aging modulating differentiation in a manner independent of conventional p53 pathways. An additional mechanism to explain the function of p53 in stem cell aging derives from its activity to maintain the genome stability necessary to prevent the aging process and/or to eliminate the damaged cells. Thus, *p53* deletion impairs clearance of chromosomal‐instable stem cells in aging telomere‐dysfunctional mice (Begus‐Nahrmann *et al*., 2009). This process is likely to be mediated by p21^CIP^ and Puma, p53 targets, which represent cooperating checkpoints limiting self‐renewal and chromosomal instability of stem cells in response to telomere dysfunction (Sperka *et al*., 2011). In support of this idea, the constitutive expression of *TERT*, concomitant with the extra *Ink4/Arf* and *p53* transgenes in mice, extends longevity, not only median but also maximum lifespan, together with a delayed exhaustion of epidermal stem cells (Tomas‐Loba *et al*., 2008). These results demonstrate that constitutive and regulated expression of p53 exerts anti‐aging activity in the context of mammalian organisms maintaining stem cell function through different mechanisms.

## Conclusion

Increasing evidence in humans and mice indicates that cancer and aging might be considered as two different manifestations of the accumulation of cellular damage, and both processes share common origins and molecular mechanisms. Among them, significant efforts have been made to determine the impact of Ink4/Rb and Arf/p53 tumor suppressor pathways on them. While their protective function against cancer is firmly established, their role in aging remains controversial.

In mice, it has been demonstrated that modest increases of regulated Arf/p53 activity are anti‐aging while deregulated activation of p53 promotes aging. These observations are not in conflict per se and indicate that the activity of Arf/p53 could be beneficial or detrimental for aging depending on their intensity and regulation. Significant amount of data has recently demonstrated that these effects are mediated through the activity of stem cells concept of a reciprocal trade between tumor suppression, aging, and stem cell biology. Based on these data, we postulate a model by which high or deregulated Arf/p53 impacts on lifespan by a decline in tissue stem cell regenerative function, but modest and regulated increases in Arf/p53 result in systemic organismal benefits ameliorating stem cell aging and maintaining tissue homeostasis. Additional work is necessary to establish the detail role and mechanism of action of p16^Ink4a^ in aging and stem cell biology.

## Funding info

This work is supported by grants from Instituto de Salud Carlos III and FEDER Funds (CP10/00539, PI13/02277), Diputacion Guipuzcoa (DFG16/002), and Marie Curie Career Integration Grant 2012/712404 to Ander Matheu.

## Conflict of interest

None declared.
